# Association of *Streptococcus Mutans, Candida Albicans* and Oral Health Practices with Activity Status of Caries Lesions Among 5-Year-Old Children with Early Childhood Caries

**DOI:** 10.3290/j.ohpd.a45411

**Published:** 2020-10-26

**Authors:** Sowmya Sridhar, Baranya Shrikrishna Suprabha, Ramya Shenoy, Ethel Suman, Arathi Rao

**Affiliations:** a Former Postgraduate Student, Department of Pediatric and Preventive Dentistry, Manipal College of Dental Sciences, Mangalore, Manipal Academy of Higher Education (MAHE), Manipal, Karnataka, India. Contributed to the study design, collection of data, writing and editing of the manuscript.; b Professor and Head, Department of Pediatric and Preventive Dentistry, Manipal College of Dental Sciences, Mangalore, Manipal Academy of Higher Education (MAHE), Manipal, Karnataka, India. Contributed to the study idea, hypothesis, design, review and editing of the manuscript with substantial contribution to discussion.; c Associate Professor, Department of Public Health Dentistry, Manipal College of Dental Sciences, Mangalore, Manipal Academy of Higher Education (MAHE), Manipal, Karnataka, India. Contributed to the study design and statistical analysis.; d Associate Professor, Department of Microbiology, Kasturba Medical College, Mangalore, Manipal Academy of Higher Education (MAHE), Manipal, Karnataka, India. Contributed to the study design and collection of data.; e Professor, Department of Pediatric and Preventive Dentistry, Manipal College of Dental Sciences, Mangalore, Manipal Academy of Higher Education (MAHE), Manipal, Karnataka, India. Contributed to the review, proofreading and editing of the manuscript.

**Keywords:** dental caries, preschool child, candida, Streptococcus mutans

## Abstract

**Purpose:**

*Candida albicans* is frequently detected together with *Streptococcus mutans* in the plaque or biofilms of children with early childhood caries (ECC). The aim of this study was to examine the association of the microbial counts of *C. albicans *and *S. mutans* in the supragingival plaque with the activity status of carious lesions and oral health practices in children with ECC.

**Materials and Methods:**

66 children aged 5 years were examined and their caries status recorded as per the ICDAS-II and the Lesion Activity Assessment (LAA) criteria. A questionnaire covering oral health practices was administered to parents. Plaque samples were collected and cultured on mitis salivarus bacitracin (MSB) agar and CHROMagar. Data was analysed using Spearman’s rank correlation and Mann-Whitney U test.

**Results:**

There was a statistically significant positive correlation between the levels of *S. mutans* and *C. albicans *(*r_s_* = 0.702, p <0.001). A positive correlation was seen between the percentage of active carious lesions with the colony counts of *S. mutans* (*r_s_* = 0.884, p <0.001) and *C. albicans *(*r_s_* = 0.785, p <0.001). Improper toothbrushing practices, dietary and feeding practices were statistically significantly associated with activity of caries lesions, *S. mutans* and *C. albicans *count.

**Conclusion:**

The total count of *C. albicans *and *S. mutans* in the supragingival dental plaque of children with ECC increases with an increase in the percentage of active carious lesions and the severity of dental caries. Improper oral health practices can lead to increased number of active carious lesions, as well as increased microbial load of both *S. mutans* and *C. albicans*.

Early childhood caries (ECC) is a chronic oral infectious disease, which if left unnoticed and untreated, has widespread repercussions not just on the oral, but also on the systemic health of the growing child.^34^ By definition, ECC refers to the presence of one or more decayed (non-cavitated or cavitated), missing (due to caries), or filled teeth surfaces in any primary tooth in a child 71 months of age or younger.^1^ A prevalence of up to 85% has been reported in developing countries.^25^

According to a systematic review, over a hundred risk factors are statistically significantly related to the prevalence or incidence of ECC, which may be classified as bacterial, behavioural or environmental.^14^ The Mutans streptococci (MS) group of bacteria has been suggested as a strong microbial risk factor in the pathogenic process of ECC.^31^ The MS group comprises of bacteria like *Streptococcus mutans* and *Streptococcus sobrinus*, with *S. mutans* being the key etiologic agent in the initiation and progression of caries.^39^

In addition to high levels of *S. mutans*, *Candida albicans*, which are otherwise commensal fungi, has been isolated at a higher frequency from the plaque bioﬁlms of children with ECC as compared to caries-free children.^27^,^33^ *Candida albicans* is known to have both aciduric and acidogenic potential and enhances biofilm formation by coaggregation with *S. mutans*.^26^

Although a number of studies^5^,^27^,^33^ including a systematic review^40^ support a positive association between oral *C. albicans *and caries experience in children, the association of *C. albicans *as well as *S. mutans* with the activity of a carious lesion is not known and needs to be studied further. Assessment of lesion activity is important for the clinician as active carious lesions progress further whereas inactive lesions often regress or get arrested.^6^ Therefore, it would be helpful to analyse the oral microenvironment to understand the factors that can potentially influence the activity status and progress of the carious lesion. In addition, although it is clear that the prevalence of *C. albicans *in children with ECC is statistically significantly higher, the effect of predisposing factors like oral hygiene and dietary habits associated with *C. albicans *carriage is relatively unknown.^40^

Thus, this cross-sectional study was undertaken to examine the association of the microbial counts of *C. albicans *and *S. mutans* in the supragingival plaque with the activity status of carious lesions in children with ECC. In addition, the effect of oral health practices on the microbial counts and activity of the caries lesions was studied. The study hypothesis was that there is a statistically significant association of the colony counts of *C. albicans *and *S. mutans* in the supragingival plaque with the percentage of active caries lesions and oral health practices of children with ECC.

## MATERIALS AND METHODS

### Sample Size and Sampling

In this cross-sectional observational study, sampling was done using cluster random sampling, with kindergarten schools as clusters. 66 children aged 5 years were selected from four randomly selected kindergarten schools of Mangalore city, Karnataka, India between December 2017–July 2018. The schools were selected by simple random sampling, using a random number table, from a list of kindergarten schools of the city. From each of the schools, only those children with voluntary written informed parental consent and fulfilling the eligibility criteria were selected. Assuming prevalence(p) of *Candida albicans* in caries-active mouth to be 60%^5^ with 10% error(d) and 90% confidence interval, the sample size was calculated to be 66. Children with at least one decayed tooth as per the definition of ECC^1^ and visible plaque on free gingival margins and adjacent surfaces of all the decayed teeth (minimum given score of 1 according to the plaque index)^35^ were included. Children with chronic systemic diseases or mentally/physically challenged children or those with history of use of antibiotics up to at least 1 month prior to the study or those on long-term regimen medication that could affect the salivary flow were excluded from the study. Ethical clearance was obtained from the institutional ethics committee prior to the study.

Parents of the selected children were asked to complete a structured close-ended questionnaire. The questionnaire covered oral health practices including oral hygiene habits such as age of initiation and frequency of toothbrushing; dietary habits such as duration of breastfeeding with history of night-time feed (child was put to sleep soon after feeding) and frequency of consumption of sugary foods. The frequency of consumption of sugary foods was recorded using a 24-hour dietary recall chart. The diet was categorised into liquid, semi-solid and solid/stick food and an overall sweet score obtained. A single sugar exposure in the form of a liquid was given a score of 5, in the form of a solid and sticky was given a score of 10 and in the form of a slowly dissolving food was given a score of 15. The cumulative score was calculated and the children were grouped into excellent, good, and watch out zone with 5 or less being ‘excellent’,10 being ‘good’ and 15 or more being ‘watch out’ zone.^28^ Prior to administration, the test-retest reliability of the questionnaire was assessed by administering the questionnaire to ten parents who were not included in the study. Retest was done one week from the day of initial administration of the questionnaire. Test-retest reliability, assessed using Cohen’s Kappa statistics, revealed a Kappa value of 0.87. The questionnaire was analysed for face validity and content validity by two subject experts and found to be satisfactory.

### Plaque Collection and Microbiological Processing

Plaque was collected using a sterile wooden toothpick that was passed around the cervical and proximal surfaces of all the teeth of the selected study participants. The tip of the wooden toothpick was then cut off and transferred to small plastic vials containing 1 ml of 0.05 M potassium phosphate buffered saline (PBS, pH-7.0). The samples were then transported within 24 h to the microbiological laboratory.

The samples were homogenised manually using a stirrer to disperse bacterial and yeast segregates. Aliquots of 0.05 ml were inoculated onto the mitis salivarus bacitracin agar (MSB agar) and CHROMagar using a sterile cotton swab, for selective recovery of *S. mutans* and *Candida albicans*, respectively. All plates were incubated at 37ºC for 48 h in an environment supplemented with 5% CO_2_. *S. mutans* was identified based on the colony morphology, as small, rough, opaque and adherent colonies.^11^ *Candida albicans* was identified by its growth on CHROMagar, based on colony appearance and colour following culture.^2^*C. albicans *was seen as pale green, spherical raised colonies. The presence of the yeast was verified further by direct microscopy (Olympus CX21i, Olympus Medical Systems India Pvt, Haryana, India) at 100x magnification of the Gram-stained smear. Thin, gram-positive spherical/oval yeast buds in chains with branching hyphae were noted.^3^ The colony counting was done by an observer blinded to the caries scores.

### Clinical Examination

Five children per day from the inclusion sample were examined in the school premises after plaque sample collection. Dental caries status of the children was recorded following the ICDAS-II criteria.^17^ All examinations were done by a single examiner who was trained using the ICDAS e-learning programme available at the International Caries Classification and Management System (ICCMS) website.^16^ As a part of the training, 10 children were examined by the examiner and rechecked by an expert examiner for reproducibility. Clinical photographs were taken, the criteria were discussed and a consensus score was given. During the study, inter and intraexaminer reliability was checked for every tenth child. Intra and interexaminer reliability was assessed using Cohen’s Kappa statistics and Kappa values of 0.88 and 0.87, respectively, were obtained.

All examinations were done using mouth mirror, gauze, WHO probe and focusable flashlight (artificial light source). The child was made to lie down on a table, while the examiner was positioned behind the child’s head. The teeth were cleaned with a wet gauze pad and then dried using cotton pellets prior to examination. The ICDAS-II scores were classified based on their severity as enamel lesions (code 1–3) and dentinal lesions (code 4–6).^7^ The scores were recorded by a trained dental assistant.

The presence of active and inactive carious lesions was assessed using the supplemental Lesion Activity Assessment (LAA) criteria. These criteria are a sum of three clinical parameters associated with the lesion: (i) clinical appearance (ICDAS-II), differentiated as any brown lesion, any white lesion or cavitated lesion [Score 1 = brown lesion (ICDAS1, 2), Score 3 = white lesion (ICDAS 1, 2), Score 4 = lesion with surface discontinuity, undermining shadow and frank cavitation (ICDAS 3, 4, 5 or 6)]; (ii) if the lesion is in a plaque stagnation area or not (Score 1 = non-plaque stagnation site, Score 3 = sites of plaque stagnation. Fossae or fissure where a ball-ended probe could enter; 0–0.4 mm from gingival margin as measured by the ball-ended probe on buccal and lingual surfaces; between the contact area and gingiva at the proximal surfaces were defined as plaque stagnation sites); and (iii) if the lesion is rough/soft or smooth/hard when a ball-ended probe is gently drawn across it (Score 2 = smooth or hard surface, Score 4 = rough or soft surface). Each surface was evaluated based on all the three criteria and a total of scores of the three parameters was taken. Any lesion with a score of 4–7 was described as ‘caries inactive’. Lesions with a sum of greater than 7 were described as ‘active’.^6^ The percentage of active caries lesions for every child was then calculated by adding the total number of surfaces with active carious lesions divided by the total number of decayed surfaces. An overall measure as to the caries status of the patient could not be obtained by the ICDAS-II criteria, as it is a surface/tooth-based evaluation. Hence, the ICDAS Caries Index (ICDAS-CI) was used to determine the caries experience, which was calculated by adding the ICDAS scores of all the decayed surfaces divided by the total number of decayed surfaces in a child.^7^

All the children with active caries lesions and in need of treatment were referred to the clinic and a chart with their treatment needs was given to the parents.

### Statistical Analysis

Statistical data was analysed using IBM SPSS Statistics for Windows, Version 20 (IBM, Armonk, NY, USA). The correlation of the colony counts of *S. mutans* with *Candida albicans* was evaluated using the Spearman’s rank correlation test. The correlation of the percentage of active carious lesions, the total number of surfaces with enamel caries (ICDAS score 1–3) and dentinal caries (ICDAS score 4–6) with the colony counts of *S. mutans* and *Candida albicans* was also done using the Spearman’s rank correlation test. The variation in the colony counts of *S. mutans*, *Candida albicans*, percentage of active carious lesions with the oral health practices (data obtained through the questionnaire) was evaluated using the Mann-Whitney U test. For all the tests, statistical significance was reported at a p value of less than 0.05.

## RESULTS

The sample consisted of 39 females, 27 males. *S. mutans* was seen to be present in all the 66 (100%) samples. *Candida albicans* was found in 34 (51.5%) children of the sample. The mean total count of *S. mutans* and *C. albicans *for the sample was 7.62 ± 7.75 × 10^3^ CFU/ml and 1.63 ± 2.23 × 10^3^ CFU/ml, respectively. There was no statistically significant difference in the percentage of active carious lesions between males and females (p = 0.932). The mean percentage of active carious lesions was 61.52 ± 26.91. The total number of children with enamel caries surfaces (ICDAS score 1–3) in the sample were 59 (89.4%) and 66 (100%) had dentinal caries surfaces (ICDAS score 4–6). The total number of enamel carious surfaces was lower (mean 3 with a minimum of 0 and maximum of 10) than the total number of carious surfaces with dentinal lesions (mean 15 with a minimum of 12 and maximum of 44). The mean ICDAS-CI value for the study sample was 4.59 ± 0.79 with a minimum of 2.14 and a maximum of 5.98. The mean dmfs was 19.78 ± 12.96 and 81.8% (54) children had severe ECC (S-ECC).

There was a statistically significant positive correlation between the colony forming units of *S. mutans* and *C. albicans *(correlation coefficient (*r_s_*) = 0.702, p <0.001). Similarly, a statistically significant positive correlation between *S. mutans* and *C. albicans *count was also seen among children with S-ECC (correlation coefficient (*r_s_*)= 0.682, p <0.001) There was a statistically significant, high positive correlation between the percentage of active carious lesions and the colony forming units of *S. mutans* and *C. albicans*. Similar results were seen with ICDAS-CI score. The number of dentinal lesions (ICDAS-II scores 4–6) showed a statistically significant positive correlation with the colony counts of *S. mutans* and *C. albicans*. No statistically significant correlation was seen for the number of surfaces with enamel caries (ICDAS-II score 1–3) with *C. albicans *count but was statistically significant for *S. mutans* (Table 1).

**Table 1:** Correlation of severity of dental caries with *S. mutans* and *Candida albicans* colony counts (×10^3^CFU/ml)

		S. mutans	Candida albicans
Percentage of active carious lesions	Correlation coefficient (r_s_)	0.778	0.750
	p value	<0.001^*^	<0.001^*^
	N	66	66
ICDAS-CI	Correlation Coefficient (r_s_)	0.636	0.645
	p value	<0.001^*^	<0.001^*^
	N	66	66
Total number of enamel caries surfaces (ICDAS scores 1–3)	Correlation coefficient (r_s_)	0.319	0.191
	p value	0.009^*^	0.124
	N	66	66
Total number of dentinal caries surfaces (ICDAS scores 4–6)	Correlation coefficient (r_s_)	0.875	0.761
	p value	<0.001^*^	<0.001^*^
	N	66	66

*p <0.05: statistically significant.

The mean percentage of active carious lesions and the colony counts of *S. mutans* and *C. albicans *was statistically significantly higher in the children who had a delayed age of initiation of toothbrushing (>1 year old) and had a brushing frequency limited to only once per day. Those children who had been breastfed, for a duration of greater than 1 year of age, with a history of night-time feed with formula milk/juice/milk with sugar were found to have a statistically significantly higher mean percentage of active carious lesions, and were found to harbour statistically significantly higher colony counts of both *S. mutans* and *C. albicans.* As assessed using the sweet score, most of the children were found to be in the ‘Watch out’ zone and had a statistically significantly higher percentage of active carious lesions, colony counts of *S. mutans* and *C. albicans *as compared to the children with a sweet score of ‘Good’ (Tables 2, 3 and 4). The association of the microbial counts with S-ECC was analysed using Mann–Whitney U test and was statistically significant for both *S. mutans* (p <0.001) and *C. albicans *(p = 0.001) count.

**Table 2:** Variation in plaque *S. mutans* colony counts (×10^3^CFU/ml) based on oral health practices

	Variable	N (%)	Minimum	Maximum	Median	Mean	Std. Deviation	Mann-Whitney U	df	p value
**Oral hygiene practices**	**Frequency of toothbrushing**									
	Once a day	47 (71.2)	0.72	23.76	4.94	9.52	8.10	185.50	1	<0.001^*^
	Twice or more a day	19 (28.8)	0.50	18.24	1.57	2.93	4.05			
	**Age of initiation of toothbrushing**									
	Before 1 year	21 (31.8)	0.50	18.67	1.56	3.36	4.72	192.00	1	<0.001^*^
	After 1 year	45 (68.2)	0.76	23.72	4.94	9.61	8.11			
**Diet and feeding habits**	**Duration of breastfeeding**									
	Less than 1 year of age	22 (33.3)	0.50	7.23	1.26	1.94	1.59	114.00	1	<0.001^*^
	More than 1 year of age	44 (66.7)	0.72	23.76	5.61	10.47	8.05			
	**Night-time feeding**									
	No	44 (66.7)	0.50	22.14	2.45	4.54	5.82	129.00	1	<0.001^*^
	Yes	22 (33.3)	2.44	23.76	17.52	13.80	7.52			
	**Sweet score**									
	Good	25 (37.9)	0.50	4.73	1.28	1.90	1.22	112.50	1	<0.001^*^
	Watch out zone	41 (62.1)	0.76	23.76	8.50	11.11	7.99			

*p <0.05: statistically significant.

**Table 3:** Variation in plaque *C. albicans *colony counts (×10^3^CFU/ml) based on oral health practices

	Variable	N (%)	Minimum	Maximum	Median	Mean	Std. Deviation	Mann-Whitney U	df	p value
**Oral hygiene practices**	**Frequency of toothbrushing**									
	Once a day	47 (71.2)	0.00	7.26	1.18	2.18	2.35	218.50	1	0.001^*^
	Twice or more a day	19 (28.8)	0.00	4.53	0.00	0.27	1.04			
	**Age of initiation of toothbrushing**									
	Before 1 year	21 (31.8)	0.00	4.08	0.00	0.32	0.98	238.00	1	0.001^*^
	After 1 year	45 (68.2)	0.00	7.26	1.18	2.24	2.39			
**Diet and feeding habits**	**Duration of breastfeeding**									
	Less than 1 year of age	22 (33.3)	0.00	0.52	0.00	0.03	0.11	165.50	1	<0.001^*^
	More than 1 year of age	44 (66.7)	0.00	7.26	2.07	2.43	2.35			
	**Night-time feeding**									
	No	44 (66.7)	0.00	7.26	0.00	1.08	1.97	280.00	1	0.003^*^
	Yes	22 (33.3)	0.00	6.20	2.84	2.73	2.35			
	**Sweet score**									
	Good	25 (37.9)	0.00	2.10	0.00	0.23	0.60	205.00	1	<0.001^*^
	Watch out zone	41 (62.1)	0.00	7.26	2.12	2.48	2.43			

*p <0.05: statistically significant.

**Table 4:** Variation in percentage of active carious lesions based on oral health practices

	Variable	N	Minimum	Maximum	Median	Mean	Std. Deviation	Mann–Whitney U	df	p value
**Oral hygiene practices**	**Frequency of toothbrushing**									
	Once a day	47 (71.2)	0.00	93.62	82.61	69.11	25.09	194.00	1	<0.001^*^
	Twice or more a day	19 (28.8)	16.67	87.50	40.00	42.75	22.00			
	**Age of initiation of toothbrushing**									
	Before 1 year	21 (31.8)	16.67	85.29	40.00	43.69	23.18	193.00	1	<0.001^*^
	After 1 year	45 (68.2)	0.00	93.62	82.61	69.84	24.56			
**Diet and feeding habits**	**Duration of breastfeeding**									
	Less than 1 year of age	22 (33.3)	0.00	73.68	30.95	38.62	20.86	131.50	1	<0.001^*^
	More than 1 year of age	44 (66.7)	14.29	93.62	84.41	72.97	21.90			
	**Night-time feeding**									
	No	44 (66.7)	0.00	87.50	57.50	52.11	26.21	156.00	1	<0.001^*^
	Yes	22 (33.3)	25.00	93.62	85.50	80.33	16.66			
	**Sweet score**									
	Good	25 (37.9)	0.00	90.00	28.57	40.81	23.68	153.00	1	<0.001^*^
	Watch out zone	41 (62.1)	20.00	93.62	84.61	74.14	20.22			

*p <0.05: statistically significant.

The data on microbial counts were recoded as presence/absence of *C. albicans *and *S. mutans* count ≥/< 10^4^ CFU/ml to obtain dichotomous categorical variables. A multivariate linear model analysis was carried out with various risk factors as independent variables (as given in Table 5) with presence of *C. albicans* and *S. mutans* above 10^4^ CFU/ml as predictor variables. Percentage of active caries lesions, caries experience as determined by ICDAS-CI and sweet score were the statistically significant independent variables associated with presence of *C. albicans* while the number of surfaces with dentinal caries was the statistically significant independent variable associated with *S. mutans* levels ≥ 10^4^ CFU/ml when controlled for other risk factors (Table 5).

**Table 5:** Multivariate Linear regression analysis with presence of *C. albicans *and* S. mutans *(≥ 10^4^ CFU/ml) as predictor variables

Variables		*S. mutans count* ≥ 10^4^CFU/ml		Presence of *C. albicans*	
		F	p value	F	p value
**Oral hygiene practices**	**Frequency of toothbrushing**	0.036	0.849	3.121	0.083
	**Age of initiation of toothbrushing**	0.903	0.346	0.67	0.796
**Diet and feeding habits**	**Duration of breastfeeding**	0.084	0.773	1.057	0.308
	**Night-time feeding**	0.392	0.534	1.522	0.223
	**Sweet score**	0.609	0.438	4.291	0.043^*^
**Severity of caries lesion**	**Total number of enamel caries surfaces** **(ICDAS scores 1–3)**	1.901	0.173	2.215	0.142
	**Total number of dentinal caries surfaces** **(ICDAS scores 4–6)**	41.481	<0.001^*^	0.073	0.789
**Activity status**	**Percentage of active carious lesions**	3.370	0.072	14.952	<0.001^*^
**Caries status**	**ICDAS-CI**	0.467	0.497	4.135	0.047^*^

## DISCUSSION 

The statistically significant positive correlation seen between the activity status of carious lesions (as measured by the percentage of active carious lesions) and the colony counts of *S. mutans* and *C. albicans *is primarily because of the ability of both the microorganisms to survive in and promote formation of acidic environment. *S. mutans* cells possess several virulence traits that affect the onset and progression of caries, such as the rapid utilisation of sucrose by cellular glucosyltransferases [Gtfs] to produce exopolysaccharides (EPS), which are the prime building blocks of cariogenic biofilm. At the same time, *S. mutans* produces acids as by-products of sugar metabolism, creating acidic microenvironments within the bioﬁlm that further select for the growth of these organisms.^36^ On the other hand, *C. albicans *can influence the activity of carious lesions, due to its own acidogenic and aciduric ability as well as synergistic activity with *S. mutans*.^40^ *C. albicans *generates acidic products through fermentation of dietary carbohydrates.^20^ In the acidic environment, extracellular enzymes (such as aspartyl proteinases), which are considered one of the most important virulence factors of this fungus become activated.^4^

*S. mutans* was found in all the plaque samples (100%) in this study. Earlier studies have shown that *S. mutans* is the most frequently found MS in the plaque of children with ECC followed by *S. sobrinus* and other species,^5^,^19^ however, none of them reported a 100% prevalence of *S. mutans* among children with ECC. This variation could be influenced by factors such as maternal *S. mutans* levels, oral hygiene, and dietary factors, salivary and immunological factors which are known to influence *S. mutans* colonisation.^21^

*Candida albicans* was found in 34 (51.5%) children of the sample. This result is in accordance with earlier studies, with oral candidal detection rates ranging 50–89% in children with early childhood caries^33^,^40^ versus 2–22% in caries-free children.^5^ Most earlier studies have reported only on the prevalence oral *C. albicans *in children with ECC.^27^,^33^ Very few studies^38^,^41^, including this study, have quantified the *C. albicans *carriage.

A statistically significant positive correlation between the colony counts of *S. mutans* and *Candida albicans* was found. This finding is consistent with the results of previous studies which suggested that *C. albicans *and *S. mutans* are found together and interact in conditions conducive to ECC.^5^,^9^ Laboratory studies have shown that *C. albicans *and *S. mutans* share a symbiotic and synergistic relationship. By virtue of its dimorphic nature, *C. albicans *is known to create a dense matrix of hyphae, which enmesh more viable *S. mutans* cells, such that the cospecies biofilm accrues more biomass.^26^ A scanning electron microscopy study revealed that the bacterial glucosyl transferases (Gtfs ) are adsorbed onto the *C. albicans *cells, producing large amounts of glucan on the fungal surface. These glucans formed in situ provide enhanced binding sites for *S. mutans* while simultaneously enhancing fungal adhesion to saliva-coated hydroxyapatite surfaces.^18^ The synergistic role of *S. mutans* and *C. albicans *shown in earlier *in vitro* studies^13^,^26^ is further strengthened by the positive correlation demonstrated between the levels of microorganisms in this cross-sectional study.

A statistically significant positive correlation was also seen between the severity of dental caries as measured by the total number of enamel and dentin carious lesions using the ICDAS-II index and the colony counts of *S. mutans* and *C. albicans.* While *S. mutans* was significantly associated with both enamel and dentinal caries, the statistically significant association of *C. albicans *count with dentinal caries, but not enamel caries, suggests that *C. albicans *has an important role in caries progression. The ability to adhere to salivary pellicle on the tooth surface with help of adhesins followed by cellular accumulation due to glucans formed in the presence of sucrose explains the higher count of *S. mutans* in the enamel lesions.^21^ Though other bacteria may have a role in initiation of early lesions, the number of *S. mutans* has been found to be higher in white spot and enamel lesions when compared to non-carious tooth surfaces.^5^,^36^ Similar to the results of this study, earlier studies have supported that *C. albicans *detection rate is positively correlated with ECC severity in terms of dmft.^22^,^41^ Prolonged acidic conditions prevail in cavitated lesions due to non-clearance of acids produced by the bacteria which promote the growth of highly acid tolerant bacteria such as *S. mutans*. Thus most of the cavitated lesions remain active.^37^

Another objective of the study was to ascertain the influence of the oral health practices on the severity of dental caries and microbial counts of *S. mutans* and *C. albicans.* It was seen that majority of the children participating in the study had started toothbrushing only after 1 year of age and brushed once a day, contrary to the current recommendations by the AAPD.^1^ According to an earlier study, factors such as increased frequency of toothbrushing, use of toothbrush and fluoridated dentifrice are known to be the protective factors against the risk of ECC.^12^ Twice-daily toothbrushing is statistically significantly more effective in maintaining oral health than brushing once a day.^29^ The results of this study show that improper oral hygiene habits can increase the streptococcal and candida counts in plaque. This could probably be due to the greater accumulation of dental plaque and creation of a mature biofilm as a result of poor mechanical plaque control, that could create an environment conducive to the co-infection and synergistic action of both *S. mutans* and* C. albicans.*^27^

In this study, those children with improper feeding and dietary practices were found to have a higher mean percentage of active carious lesions and higher colony counts of *S. mutans* and* C. albicans.* Breastfeeding beyond 24 months and *ad libitum* feeding have been established as important risk factors in the development of ECC.^1^ Because of the poor oral clearance of oral carbohydrates during night-time feeding (the child is put to sleep soon after feeding), especially with sweetened milk or juice, an environment conducive to growth of pathogenic microorganisms is created.^8^ In regard to ECC risk and overall cariogenicity of the diet, it is the amount of time that the oral cavity is exposed to fermentable carbohydrates, rather than the total amount consumed, that is the most critical factor to be considered.^30^ In our study, most of the children had a sweet score belonging to the ‘Watch out’ zone and also had higher percentage of active carious lesions and colony counts of *S. mutans* and *C. albicans*. These findings are consistent with an earlier study, which showed that the odds for ECC and the colony counts of cariogenic microflora were greater in children with a higher total sugar exposure.^31^ Similarly, oral candida carriage has been seen to increase in the presence of sucrose and cause an increase in the rate of formation of occlusal caries since the adhesive interaction between *S. mutans* and *C. albicans *is enhanced.^20^ Thus in this study population, feeding and dietary practices which were conducive for ECC also contributed to increased activity and severity of carious lesions. Considering these outcomes, it is important to educate parents of children with ECC, regarding the role of improper oral health practices related to oral hygiene, feeding patterns and sucrose consumption in the diet to decrease the microbial count of *S. mutans* and *C. albicans*, which in turn will decrease the severity and activity of caries lesions in children with ECC. It should be emphasised during parent education that, implementing proper oral health practices not only prevent new carious lesions but also decrease the activity of the existing carious lesions and prevent their progression.

The results of the study suggest the association of multiple factors such as oral health practices, severity of caries lesion, activity status of the caries lesions with the microbial count of *S. mutans* and *C. albicans*. To adjust the intereffect between the variables, a multivariate analysis was carried out. The results show that among the oral health practices, the number of sugar exposures in the diet is the most important determinant of *C. albicans*, along with caries lesion activity and caries experience, while higher number of dentinal caries lesions is a determinant of *S. mutans* levels. While earlier studies have shown the association of oral candida carriage with increased presence of sucrose in the diet^20^ as well as increased ECC severity in terms of dmft^41^, the results of the study confirm the same. In addition, the association of *C. albicans *with the activity status of the caries lesion is shown. Also, the association of *S. mutans*, which is a known risk factor of ECC^31^, with the severity of caries lesions is shown by this study. This underlines the need for proper identification and treatment of active caries lesions, along with diet counselling to bring about decrease in microbial counts.

In this study, children of the same age (5 years) were selected as age is known to be a confounding factor for caries experience.^24^ Plaque samples were collected since we expected a greater probability of detection of *Candida albicans* from plaque based on earlier studies, as opposed to salivary samples or oral swabs.^10^ The variation in secretion rate and duration of saliva contact with biofilm on enamel surfaces can lead to variability and lack of reproducibility. Also, saliva samples may be non-representative of the microbial profile at the sites where dental caries take place.^15^

The caries experience of the child was evaluated using the International Caries Detection and Assessment System (ICDAS-II) and its subset, the LAA criteria instead of the dmfs index. Although commonly used, the dmfs index is unable to differentiate between enamel and dentinal lesions and give reliable data regarding the possible progression of the lesion based on activity.^7^ Most of the caries lesions were active resulting in high mean percentage (around 60%) of active caries surfaces per individual. The proportion of number of dentinal caries surfaces was much higher than enamel surfaces. Most of the dentinal lesions are cavitated and act as plaque stagnation areas which increases the microbial load and thereby the activity of the lesion.^32^

One limitation of the study was the use of a cross-sectional study design through which only association of predisposing factors with the disease can be studied and the temporal nature of the cause and effect cannot be ascertained.^23^ Therefore, with such a study design, it is difficult to conclude if the presence of high levels of *S. mutans* and *Candida albicans* contributed to caries progression or whether high levels of *S. mutans* and *Candida albicans* was due to the presence of active caries lesions. Hence further prospective cohort studies need to be carried out in order to better understand the predisposing factors for active carious lesions.

In spite of the limitation, the results obtained in this study demonstrated a quantitative association of *Candida albicans* and *Streptococcus mutans* in the supragingival plaque with the activity status and severity of carious lesions in children with ECC, as well as the influence of oral hygiene and dietary practices on these parameters. The results suggest that both the microbes have an important role in caries progression. Based on this, further research could be directed at the combined use of antifungal and antibacterial therapies in caries control. Considering the association of the levels of *C. albicans *with *S. mutans*, further research may be directed at anti-adhesive strategies aimed at blocking this interaction to modify the caries experience.

## CONCLUSIONS

Within the confines of the study, it can be concluded that:

1. The colony counts of both *Candida albicans* and *Streptococcus mutans* in the supragingival dental plaque of children with ECC increase with an increase in the percentage of active carious lesions and the severity of dental caries.
2. Oral health practices like initiation of toothbrushing after 1 year of age, not brushing twice a day, prolonged night-time breast/bottle feeding, and frequent sugar consumption in the diet can result in increased number of active carious lesion as well as increased microbial load of both *S. mutans* and *C. albicans *in the supragingival plaque.
3. Increased sugar exposure in the diet, caries experience and activity of caries lesions are the most important factors associated with *C. albicans *while higher number of dentinal caries lesions is a determinant of *S. mutans* levels.
